# The contribution of dietary restriction to extended longevity in the malaria vector *Anopheles coluzzii*

**DOI:** 10.1186/s13071-017-2088-6

**Published:** 2017-03-24

**Authors:** Roy Faiman, Samantha Solon-Biet, Margery Sullivan, Diana L. Huestis, Tovi Lehmann

**Affiliations:** 1 Laboratory of Malaria and Vector Research, NIAID, NIH, Rockville, MD 20852 USA; 2 Charles Perkins Centre, University of Sydney, Sydney, 2006 Australia; 3 Office of Global Health Diplomacy, U.S. Department of State, 1800 G Street NW, Suite 10300, Washington, DC 20006 USA

**Keywords:** *Anopheles coluzzii*, Aestivation, Dietary restriction, Longevity, Dry season

## Abstract

**Background:**

Variation in longevity has long been of interest in vector biology because of its implication in disease transmission through vectorial capacity. Recent studies suggest that *Anopheles coluzzii* adults persist during the ~7 month dry season *via* aestivation. Recently there has been a growing body of evidence linking dietary restriction and low ratio of dietary protein to carbohydrate with extended longevity of animals. Here, we evaluated the effects of dietary restriction and the protein : carbohydrate ratio on longevity of *An. coluzzii*.

**Results:**

In our experiment, we combined dietary regimes with temperature and relative humidity to assess their effects on *An. coluzzii* longevity, in an attempt to simulate aestivation under laboratory conditions. Our results showed significant effects of both the physical and the dietary variables on longevity, but that diet regimen had a considerably greater effect than those of the physical conditions. Higher temperature and lower humidity reduced longevity. At 22 °C dietary protein (blood) shortened longevity when sugar was *not* restricted (RH = 85%), but extended longevity when sugar *was* restricted (RH = 50%).

**Conclusions:**

Dietary restriction extended longevity in accord with predictions, but protein : carbohydrate ratio had a negligible effect. We identified conditions that significantly extend longevity in malaria vectors, however, the extent of increase in longevity was insufficient to simulate aestivation.

**Electronic supplementary material:**

The online version of this article (doi:10.1186/s13071-017-2088-6) contains supplementary material, which is available to authorized users.

## Background

Although significantly reduced in the past decades, malaria transmission still impacts humanity heavily, with annual mortality near half a million, most victims are children in sub-Saharan Africa [1]. *Anopheles coluzzii* (formerly *An. gambiae* M-form), *An. gambiae* (*sensu stricto*) (*s.s*) (formerly *An. gambiae* S-form), and *An. arabiensis* (*An. gambiae* complex), are the dominant malaria vectors in West Africa [2] and their distribution ranging from tropical Africa to the arid Sahel [3]. *Anopheles coluzzii* bionomics markedly differs from that of *An. gambiae* (*s.s*) and *An. arabiensis* [4, 5]. Throughout the Sahel it is the only species that persists during the seven month long dry season (when no surface water is available) beginning its population growth soon after the first rains. Its other two sibling species apparently vanish during the dry season, and begin their population growth some 6–8 weeks after *An. coluzzii*, likely following reintroduction *via* migration [6–8]. The extended adult survival throughout the long dry season, referred to as aestivation, is backed by previous studies on these species [6, 7, 9–16]. This seasonal variation in longevity is of considerable interest but despite early success in simulating it under laboratory conditions [11, 16] additional attempts failed to reproduce these results [17–19].

Seven-fold extension of adult longevity is the primary hallmark of aestivation in *An. coluzzii* [6–8, 20]. Additional key changes during the dry season include depressed reproduction [9], depressed flight activity [15], increased desiccation tolerance linked to changes in cuticular hydrocarbons [21] and metabolic and protein changes [13, 22], but not lower metabolic rate [15]. The decline in population density some four weeks before larval sites dry up [7] and the allometric changes in thorax and spiracle size [21] are also consistent with developmental and behavioral changes characteristic of the diapause-induction phase [23, 24], yet the constellation of seasonal changes in this species suggest an atypical form of dormancy [20]. For example, reduced blood-feeding rate was inferred [7, 15, 20] but not measured in indoor resting females [9]. Changes in protein/carbohydrate catabolism may be key to the induction and maintenance of aestivation as suggested, by formation of long-lived dauer-state larva in *Caenorhabditis elegans* (Maupas, 1900) (Rhabditida: Rhabditidae) carrying mutations at the insulin and IGF-I receptors [25, 26]. Similarly, in *Drosophila* Fallén, 1823 (Diptera: Drosophilidae), mutations on analogous genes also resulted with diapause and a 45–85% extension in lifespan [27–31]. Lifespan extension thus seems to be mediated through a number of nutrition-linked metabolic pathways [32], often at the cost of reproduction [33].

Adult mosquitoes are obligate sugar-feeders. A sugar meal is especially important soon after adult emergence, but also subsequently as it is the primary fuel for flight and prolongs survival after egg deposition [34, 35]. Sources of sugar for mosquitoes include floral nectar, extra-floral nectaries, and plant fluids [36–41]. Dietary-restricted (DR) organisms across many taxa have been reported to survive longer than those with unlimited or normal access to food. For example, lifespan increased 30–70% in rodents, spiders, and grasshoppers [42–48], 2-fold in budding yeast and *Drosophila* [49–52], and up to 10-fold in *C. elegans* [53, 54]. Studies on yeast, *C. elegans* and *Drosophila* showed that DR can be viewed as a Gaussian curve of food intake, situated centrally between starvation and ad-libitum food intake [55–60]. In *C. elegans,* larvae can enter a unique dormant state (“dauer”) with the onset of starvation in the early larval stages. In this state the worms can extend their lifespan significantly by reducing oxidative stress, and subsequently the rate of aging, until such times when conditions promote normal sustenance [61, 62]. Tatar & Yin [63] claim this mechanism of retarded aging during reproductive diapause has co-evolved in several insect groups including butterflies, grasshoppers and flies. In *Drosophila*, the effect of diet on longevity depends on genetic background and is mediated by a shift in activity of superoxide dismutase (SOD) gene [50]. Aging and nutrition in *Drosophila*, are linked by protein-to-carbohydrate (P:C) ratio, resulting in the tradeoff between lifespan and reproduction [64], as predicted under the Nutritional Geometry framework (NGF) [65]. Accordingly, shifts within this ratio can either prolong life (1:16), or alternately increase egg production (1:2 or 1:4) [64]. The NGF separates the effects of calories and nutrient composition on lifespan [66] based on consistent patterns across diverse taxa, ranging from yeasts and *C. elegans* to insects, and even primates and humans [67–70].

Anautogenous female mosquitoes, unlike the majority of taxa listed above, ingest proteins and carbohydrates separately rather than in mixtures. Unlike sugar meals, which lack proteins and have very low concentrations of amino acids, blood meals, taken only by females are protein-rich although they also provide sizable portions of the lipids and carbohydrates. Although, most of the blood meal nutrients are utilized for reproduction, the carbohydrates from sugar meals taken by males and females alike provide mostly energy for sustenance.

In this sense, mosquitoes exhibit a more “digital” system of macronutrient balancing, therefore the ratio of blood meals to sugar meals provides a meaningful approximation of P:C ratio with high relevance to reproduction/sustenance polarity. It follows that experimental incorporation of P:C ratio into mosquito diets refer to nutrients offered as “parcels” of blood meal/days and sugar/days.

With the aim of exploring the effects of nutritional regimes on longevity of *An. coluzzii*, and especially on simulating aestivation under laboratory conditions, we subjected a recently established colony to an array of nutritional regimes, coupled with temperature and RH conditions characteristic of the Sahelian dry and rainy seasons. We hypothesized that both dietary restriction (reduction in either sugar and/or blood intake), as well as reduction in P:C ratio would prolong mosquito lifespan, especially when coupled with lower temperature and RH.

## Methods

Two separate experiments were carried out using the *An. coluzzii*, Thierola strain, which was established from six wild-caught females that were collected in Thierola, Mali (13°39'31"N, 7°12'54"W) in November 2012. Eggs, and later larvae were reared per standard procedures [71]. In each of the two experiments, two batches of four trays, each with 330 eggs were placed in plastic trays (30 × 25 × 7 cm) with 300 ml dechlorinated tap water. In the first experiment, trays of larvae were kept at constant conditions of 27 °C, 85% RH (either at 12:12 or 11:13 L:D, with 1 h of dusk and dawn). In the second experiment, all eggs and larvae were hatched and reared under short photoperiod conditions. During the first two days after hatching began, a 2 ml yeast slurry (2 mg dry yeast in 50 ml water) was added to trays (in both experiments), then finely-ground Koi fish food (38% protein, 13% ash) (Optimum Hi Pro, Perfect Companion Group Ltd., Samutprakarn, Thailand) was added daily until pupation 8–10 days after hatching.

Groups of 40–45, 1-day-old females were caged inside half-gallon plastic cages, together with 20 males per cage. Mating was allowed to take place over a 5-day period at 27 °C and 85% RH, with 25% honey (Oaxaca organic honey, Kevala®, Dallas, TX, USA) *ad libitum*, after which the males were removed. At this point, insemination rate was 83 and 82% for the first and second experiments respectively. The first blood meal was provided on day 5 using a water-heated glass feeder at 38 °C with a hog-gut membrane and bovine blood (Cat. no.7200804; whole bovine blood with CPD anticoagulant, Lampire Biological Laboratories, Pipersville, PA, USA). On the following day, blood-fed females only were allocated randomly into four treatments: low temperature (22 °C and 23.5 °C in first and second experiments respectively); high RH (85%) and low RH (50%), and standard rearing temperature (27 °C); low and high RH (Table 1). Low RH conditions were achieved by placing cages in hermetically- sealed translucent plastic containers (50 × 35 × 18 cm, Iris USA Inc., Pleasant Prairie, WI, USA) with 250 ml of a super-saturated desiccant salt solution (MgCl or LiCl, Sigma-Aldrich, St. Louis, MO, USA). A miniature 5 Volt DC brushless fan (Model: FSY42S05H, Shenzhen FengShengYuan Electronics Co. Ltd., Shenzhen, China) affixed above the desiccant solution container provided air circulation and uniform air desiccation within the low RH chamber (Additional file 1: Figure S1). A digital thermometer/hygrometer (Model: Traceable™, Fisher-Scientific™, Pittsburgh, PA, USA) was used to monitor temperature and RH within the box. Sugar in the form of a 25% organic honey solution was provided through a 1 mm-thick cotton wick (No.1/0 square braid, PremiumCraft™, Wicks) protruding 1 cm out of a 25-ml glass beaker, and secured with Parafilm™ to prevent mosquitoes from entering the beaker and to minimize water vapor release into the cage. Honey proved somewhat superior for longevity compared with several concentrations of sucrose, fructose or glucose tested beforehand (Faiman et al., unpublished). Although African honey from the Sahel would be preferred, organic honey (from Mexico) was chosen instead of sugar because it contains electrolytes and micronutrients that naturally occur in nectar and may have a role in long term longevity [40, 72]. Blood and sugar access were arbitrarily provided per treatment on different schedules as described below (Table 2). Based on our insectary blood-feeding schedule of once per 7 days, this interval was selected as the standard for non-restricted blood diet [73, 74]. Blood meals took place between 5–6 pm in a darkened room at 27 °C, 80% RH. All treatment groups were allowed 30 min to acclimatize to the feeding room conditions before blood was offered. Mosquitoes were allowed to feed for 15 min and were given an additional 5 min if the feeding rate was < 50%, after which treatment cages were returned to their assigned conditions. Paper cups with 50 ml tap water and a filter paper (No.1 Whatman™, Buckinghamshire, UK) cone on top (as oviposition substrate) were provided only to wet season treatments (85% RH) for oviposition, 48 h after the blood meal, and were removed 48 h later. Dry-season treatments (50% RH) were not provided with oviposition water, i.e. were subjected to oviposition site deprivation [74]. Handling and maintenance of the cages was minimized as much as possible to reduce human interference and subsequent stress in the mosquitoes. All treatments received similar handling, whether for maintenance (honey change, blood meal, etc.) or mock maintenance in cages where no maintenance was required.

**Table 1:** Experimental setup of the two block-like experiments. Temperature and relative humidity correspond to either early or late rain or dry seasons. Photoperiod in Experiment 2 was set to DS conditions

	Early RS	Late RS	Early DS	Late DS
Experiment 1				
Photoperiod (L:D)	11:13	12:12	11:13	11:13
Temperature (°C)	22	27	22	27
RH (%)	85	85	50	50
Experiment 2				
Photoperiod (L:D)	11:13	11:13	11:13	11:13
Temperature (°C)	23.5	27	23.5	27
RH (%)	85	85	50	50

*Abbreviations*: *LD*, Light: Dark; *RH*, relative humidity; *RS*, rainy season; *DS*, dry season

**Table 2:** Diet regime in each of the two experiments. Dietary sugar is shown as days of sugar available/7- days. Dietary blood is shown as frequency of blood meal available per 7, 10, 14 d, once only, or none (0)

	Experiment 1		Experiment 2	
Diet	Sugar^a^	Blood meals^b^ (index value)	Sugar^a^	Blood meals^b^ (index value)
1	7:7	1:7 (0.143)	7:7	1:10 (0.1)
2	3:7	0 (0)	2:7	0 (0)
3	3:7	1:14 (0.071)	2:7	Once (0.033)
4	1:7	0 (0)	1:7	Once (0.033)
5	1:7	1:14 (0.071)	1:7	1:10 (0.1)

^a^Sugar days: 7-day cycle

^b^Blood meal days: 7-day cycle (Experiment 1); 10-day cycle (Experiment 2)

### Experimental feeding regime

Within each of the four temperature/RH treatments, namely higher (27 °C) or lower (22 °C, 23.5 °C) temperature with higher (85%) or lower (50%) RH, five dietary regimens were provided, totaling 20 treatments (Table 2).

Adult mosquitoes subjected to each diet regime were exposed to four different climate combinations representative of Sahelian climate: (a) cool, early dry season (December-February; 22–23.5 °C, 50% RH), (b) warm, late dry season (March-May; 27 °C, 50% RH), (c) cool, early wet season (June-July; 22–23.5 °C, 85% RH) and (d) warm, late wet season (August-November; 27 °C, 85% RH) (Huestis & Lehmann [20] and unpublished). Temperature and RH levels were selected based on average nightly temperatures measured in Thierola (from 18:00 to 06:00). The 50% RH level was selected for dry season conditions assuming that unlike outdoor RH, the humidity in a presumed underground shelter is elevated.

### Statistical analysis

Relationships between longevity and underlying factors were tested using univariate survival analysis in Proc Lifetest (SAS), to test differences between levels of each factor using Kaplan Meier survival function and Wilcoxon tests. Diet regime was expressed as the frequency per week of access to either sugar or blood, separately. For example, if a meal was offered daily, once a week, or once every 10 d, the corresponding index values were 1, 0.143, and 0.1, respectively (see Tables 2 and 3). The special case where a blood meal was offered only once in lifetime (above), was arbitrarily assigned the value of 0.033 (one blood meal in a 30-day mean lifespan), whereas no blood meal throughout was assigned the value of 0. Multivariate analysis using Cox proportional hazard regression (Proc Phreg, SAS) was carried out with stratification by experiment. In the first analysis (Model 1), we evaluated the effect of diet regimes by the treatments that mosquitoes were assigned to, regardless of date of death in relation to meals offered. In this analysis mosquitoes assigned to diet of once a week blood meal and once in a life time were classified in different treatments, even if they died in the first week after the blood meal, i.e. during the period they experienced identical diets. To accommodate for the actual diet regime, we also performed a second analysis (Model 2), where meals were included as time dependent covariates. In the latter analysis, the mosquitoes in the example above were exposed to the same diet regime in the first week, but after the first group received the second blood meal, their diet treatment differed. The assumption of proportional hazard was assessed visually and tested by the supremum test (test that the observed pattern of martingale residuals is not different from the expected pattern so the model is correctly specified). This test calculates the proportion of 1,000 simulations that contain a maximum cumulative martingale residual larger than the observed maximum cumulative residual. Finally, the interaction of time with suspect variables, with respect to non-proportional hazard, was introduced into the model. Significant variable-time interactions attested against the proportional hazard of the variable alone. In which case, the interaction was retained in the model [75].

**Table 3:** The effects of temperature, RH, and nutritional regime, with interactions on longevity: Model 1, by treatment (= exposure) Cox regression, with varying conditions of temperature and relative humidity. Model 2, with time-dependent diet-regime variables and varying conditions of sugar or blood consumption

Parameter^a^	Model 1 (Treatment)^b^ -2LL = 15819.69, AIC = 15,819.69		Model 2 (Time-dependent)^c^ -2LL = 14649.21, AIC = 14661.21		Estimates of parameter effects^d^			
					Model 1		Model 2	
	Estimate (*χ* ^2^)	*P*-value^e^	Estimate (*χ* ^2^)	*P*-value^e^	Conditions	Hazard ratio (95% CI)	Conditions	Hazard Ratio (95% CI)
Temperature	0.027 (0.26)	0.610	-0.095 (3.22)	0.073	∆5 °C & 50% RH	1.28 (1.06–1.53)		
					∆5 °C & 85% RH	2.12 (1.74–2.58)		
RH	-0.054 (8.07)	**0.005**	-0.078 (16.34)	**< 0.0001**	∆10% RH & 22 °C	0.86 (0.82–0.91)		
					∆10% RH & 27 °C	1.00 (0.95–1.04)		
Sugar	-4.714 (135.35)	**< 0.0001**	-0.122 (86.45)	**< 0.0001**	BM 1/10 d, Sug 0.14	0.66 (0.47–0.93)	1 BM/2 Sug	0.35 (0.32–0.38)
					BM 1/10 d, Sug 0.33	1.35 (0.97–1.88)	1 BM/14 Sug	0.45 (0.41–0.49)
					BM 1/10 d, Sug 1	17.14 (9.57–30.69)	1 BM/50 Sug	0.94 (0.69–1.29)
Blood	-9.516 (23.88)	**< 0.0001**	-1.089 (616.51)	**< 0.0001**	Sug 1/7 d, BM 0	0.51 (0.46–0.57)	1 Sug/BL 0	0.89 (0.86–0.91)
					Sug 1/7 d, BM 0.05	0.67 (0.62–0.72)	1 Sug/BL 4	0.96 (0.95–0.97)
					Sug 1/7 d, BM 0.15	1.15 (1.10–1.21)	1 Sug/BL 9	1.07 (1.02–1.11)
Temp x RH	0.002 (5.84)	**0.016**	0.003 (13.97)	**0.0002**				
Sug*BL	37.928 (120.07)	**< 0.0001**	0.021 (31.94)	**< 0.0001**				
Time*BL	-0.379 (22.45)	**< 0.0001**	-0.380 (22.45)	**< 0.0001**				

Bold *P*-values indicate statistical significance

^a^All parameters had 1 *df*

^b^Based on allocation to treatment (= cage)

^c^Based on allocation by day of death

^d^Estimates based on condition ranges relevant to laboratory and field conditions

^e^Significant *P*-values shown in bold-face

*Abbreviations*: *BM* blood meal (1/10 = once every 10 days); *Sug* sugar (1/7 = once every 7 days); *RH* relative humidity

Median lifespan data was also analyzed using response surfaces and visualized in 2-dimentional heat maps (thin-plate splines). Response surfaces model median lifespans over sugar and/or blood meals for each of the six temperature/RH combinations. The effects of these diets were broken down to main effects and interactions. Red regions in each response surface indicate greatest values for median lifespan and blue regions indicate lowest values. Contour lines within the nutrient space display lifespan in days and reflects the specific diet combination of blood and sugar. Response surfaces were plotted as thin-plate splines with the use of the ‘*mgcv*’ package in R and analyzed using generalized additive modelling (GAM) in R (v. 3.1.3) as previously described in [76].

Throughout the analysis, we treated the two experiments as replicates, albeit the treatment factors had certain values (levels) which differed between experiments. Thus, “cage effect” was incorporated into the analysis by considering experiments as a block (strata in Cox regression terminology) and in the formulation of the model as a regression analysis (unlike ANOVA), allowing us to explore a larger “nutritional space”. In Cox regression, each value of a variable, e.g. zero-blood or daily-sugar meal may be unique but the inter-cage variance/affect determines the significance of the tested treatment-factors, i.e. blood. This allowed us to measure responses to each treatment factor without replicating each particular value of the nutritional regimes. Hence, the cage effect is incorporated into the analysis by specifying the unique combination of treatments and interactions, so each cage is specified.

## Results

Longevity of a total of 609 and 912 females was measured (130 and 15 mosquitoes were right-censored) in the first and second experiments respectively. Overall, mean longevity was higher in the first experiment (22.3 *vs* 19.9 days; Wilcoxon test: *P* = 0.0496, *χ* ^2^ = 3.86, *df* = 1).

### Longevity and climatic conditions

**Fig. 1:**
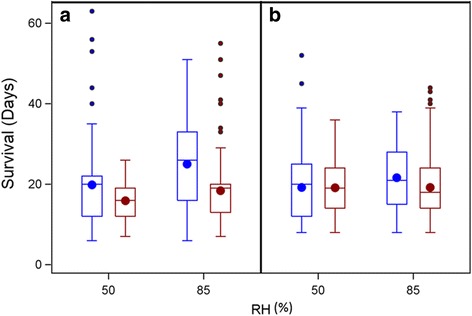
Median and mean survival by temperature and RH; Experiment 1 (**a**), 22 °C (in *blue*) and 27 °C (in *red*). Experiment 2 (**b**), 23.5 °C (in *blue*) and 27 °C (in *red*) at low (50%) and high (85%) relative humidity (RH). Circles inside boxes denote the means, horizontal line denotes median. Whiskers are 25 (bottom) and 75 (top) percentiles. Dots above whiskers are outliers. Note that low temperature in Experiment 2 was 1.5 °C higher than in Experiment 1

**Fig. 2:**
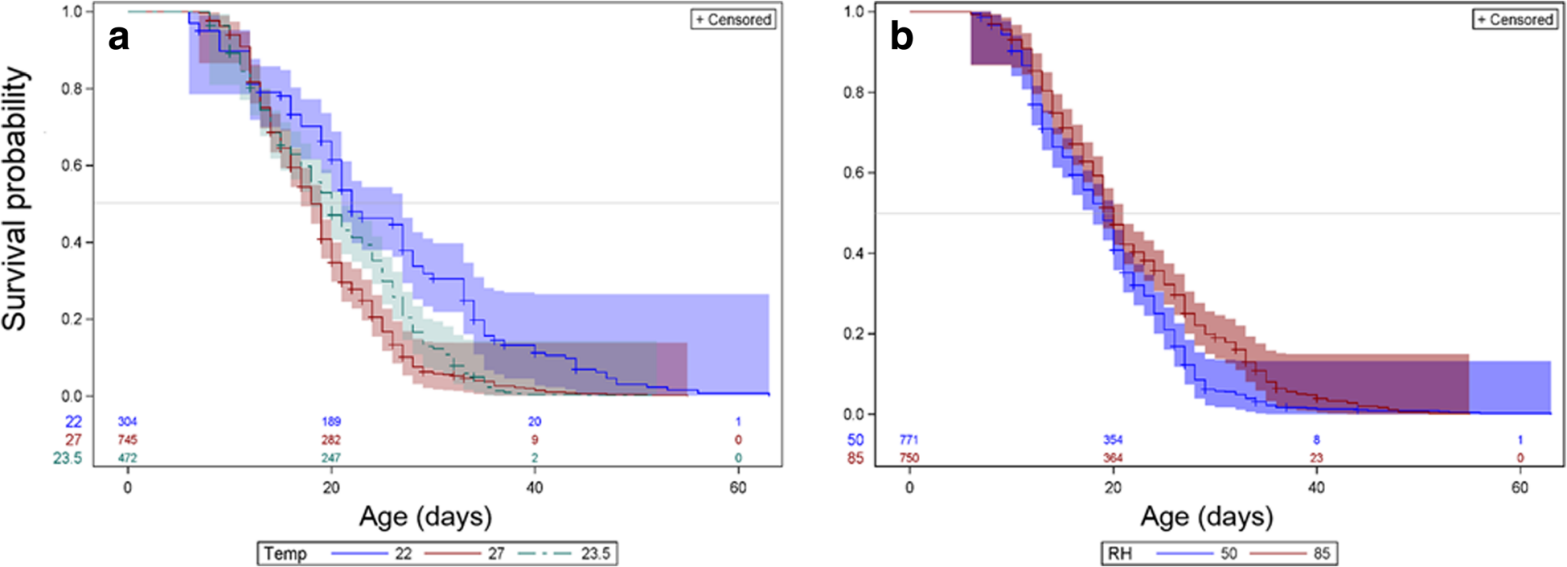
Longevity by temperature (**a**) and relative humidity (RH) (**b**); Kaplan-Meyer Product-Limit Survival curves, with number of subjects at risk (above X-axis) and 95% CI (Hall-Wellner bands). Color coding: **a** 22 °C (*blue*), 27 °C (*red*), 23.5 °C (*green*); **b** low RH (50%, *blue*), high RH (85%, *red*). 50% survival probability is marked by *gray* horizontal line

Longevity increased at lower temperature although, unlike its highly significant interaction with RH (*P* < 0.001, Table 3), the main effect of temperature was not statistically significant (*P* = 0.072, Table 3). The effect of temperature was more pronounced in higher RH (Fig. 1) and was greater in the first experiment because the low temperature (22 °C) was 1.5 °C lower than that used in experiment 2 (22 *vs* 23.5 °C, Fig. 2). Higher longevity in lower temperature was detected in each experiment as well between 22 °C and 23.5 °C (*P* < 0.001 Wilcoxon tests on Kaplan Meyer functions), but not between experiments in 27 °C (*P* = 0.072, Wilcoxon tests on Kaplan Meyer functions). Similarly, survival probability (longevity) increased at higher RH (85 *vs* 50%, *P* = 0.013 Wilcoxon tests on Kaplan-Meyer functions) and this was more pronounced in lower temperature (Fig. 2).

Multivariate Cox regression showed that higher temperature decreased survival, but in low humidity (50%), a change of 5 °C increased the hazard rate (i.e. decreased longevity) by 80% whilst in high humidity (85%), the same temperature change increased the hazard rate by 150% (keeping the other covariates at their mean values). Moreover, at a low temperature (22 °C), an increase of 10% RH decreased the hazard rate by ~13%, whilst at 27 °C, the same RH change decreased the hazard rate by ~5% (Table 3).

### Longevity and diet

**Fig. 3:**
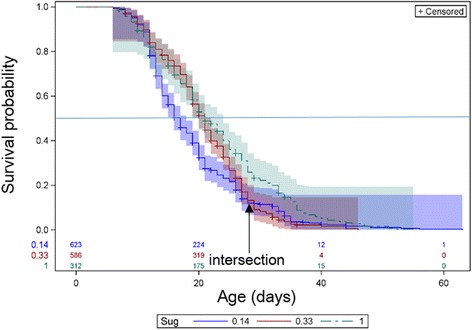
Longevity by nutritional sugar availability; Kaplan-Meyer Product-Limit Survival Estimates, with number of subjects at risk and 95% CI (Hall-Wellner bands). Color coding: one sugar day every 7 days (*blue*), 2–3 days every 7 days (*red*), *ad libitum* (*green*). 50% survival probability is marked by *gray* horizontal line

**Fig. 4:**
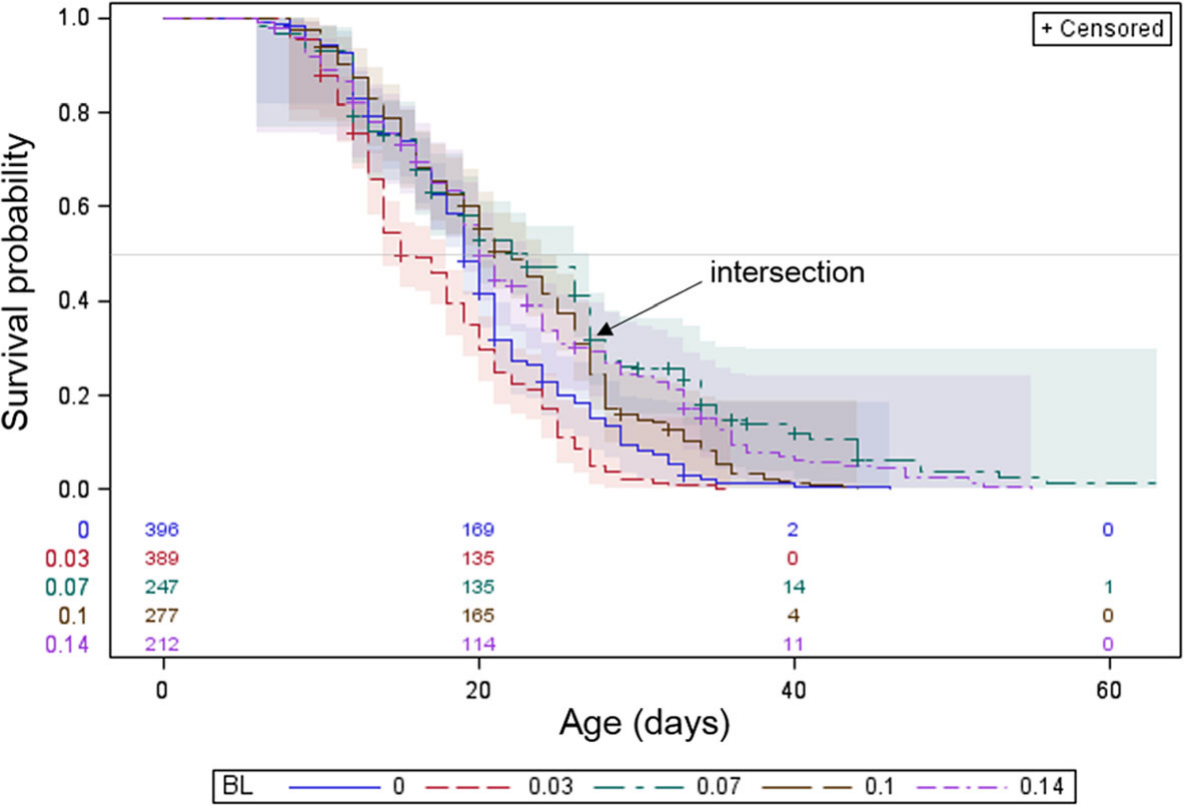
Longevity by nutritional blood availability; Kaplan-Meyer Product-Limit Survival Estimates, with number of subjects at risk and 95% CI (Hall-Wellner bands). Color coding: no blood (*blue*), once only (*red*), once every 14 days (*green*), once every 10 days (*brown*), and once every 7 days (*purple*). 50% survival probability is marked by *gray* horizontal line

The main effects of dietary sugar and blood were evaluated separately (across all other effects, Figs. 3 and 4). Overall, longevity increased with greater access to sugar (Wilcoxon test, *χ* ^2^ = 50.7, *df* = 2, *P* < 0.001), yet the effects of different moderate and low access to sugar appear to change over time as indicated by the intersection of the survival functions (Fig. 3). Significant differences were also found between mosquitoes with different access to blood meals (Fig. 4, Wilcoxon test, *χ* ^2^ = 91.3, *df* = 4, *P* < 0.001). Notably, highest longevity was attained by groups with reduced access to blood. Here too, survival functions of treatments with different access to blood meals crossed each other, indicating that the effects changed over time (Fig. 4).

Multivariate analysis using Cox regression, commonly used in survival analysis, revealed highly significant and large main effects of dietary sugar and blood, as well as a strong and significant interaction (Table 3). However, unlike the main effects, the interaction increased the hazard rate. Accordingly, adding a blood meal once every 10 days reduced the hazard rate (i.e. increased longevity) by 50% at low dietary sugar of one day a week, but the same change in dietary blood at high (daily) dietary sugar, increased the hazard rate (i.e. mortality) by 950% (see Model 1 conditions; Table 3). The significant interaction of dietary blood with time (Table 3) alluded to above, suggested that increased dietary blood decreased the hazard rate to a greater extent in older mosquitoes.

To evaluate the roles of DR and P:C ratio on longevity, the longevity hazard ratio (and 95% CI) was computed using the Cox regression model for adding a single blood meal at a range of sugar meals and vice versa (see Model 1 conditions; Table 3). Spearman correlations were calculated between the longevity odds ratio and the P:C ratio, as well as the degree of DR for both blood and sugar (holding the other constant). The Spearman correlation coefficient was negative and highly significant between the longevity odds ratio and DR (*r*_s_ = -0.94, *P* = 0.005, *n* = 6, Table 3). However, the Spearman correlation coefficient between P:C ratio and the longevity odds ratio was non-significant (*r*_s_ = -0.029, *P* = 0.922, *n* = 6, Table 3).

The analyses above (Model 1) pertaining to the individual treatments (cages) ignored the fact that per-mosquito diet regimes were time dependent, and thus, particular regimes differed from each other only after the first 10–14 days, depending on blood-meal regimen. Thus, we also analyzed the data using time dependent variables (Model 2; Table 3), in which, the cumulative number of sugar- and blood- meals a mosquito was offered (until it died), reflected her diet regime, regardless of the cage (= treatment) she was assigned to. Accordingly, a mosquito that received one blood meal every 10 days and died at day 18 had one blood meal, as was the case for one that received one blood meal every 7 days and died at day 11. The overall reduction in model fit criteria (-2LL, AIC, and SIC) indicated a better fit of the model based on time dependent diet variables. The results showed very similar significance and pattern of the main effects and the interactions detected above, although the parameters were more modest in magnitude as revealed by the hazard ratios (Model 2, Table 3). Notably, the main effects of sugar and blood and their interaction were highly significant, and unlike the main effects, the interaction increased the hazard rate. However, adding one blood meal reduced the hazard rate by 65% at low sugar access of two sugar meals, whilst adding one blood meal after 50 sugar meals reduced the hazard rate only by 6% (which was not significant from no effect, *P* > 0.05, see Model 2 conditions, Table 3). This amounts to a six-fold change in the effect size. This reduction in the magnitude of diet effects probably reflects that age confounds the diet because a mosquito that had 50 sugar meals must be at least 50 days old. Similarly, adding one sugar meal for a mosquito that had no blood meals reduced the hazard rate by 15%, whilst adding one sugar meal for a mosquito that had 9 blood meals increased the hazard rate by 1% (*P* < 0.05, see Model 2 conditions, Table 3). The two models, each with its limitations, provide complementing views of our results. We believe that the consensus between the models allows for a balanced interpretation.

**Fig. 5:**
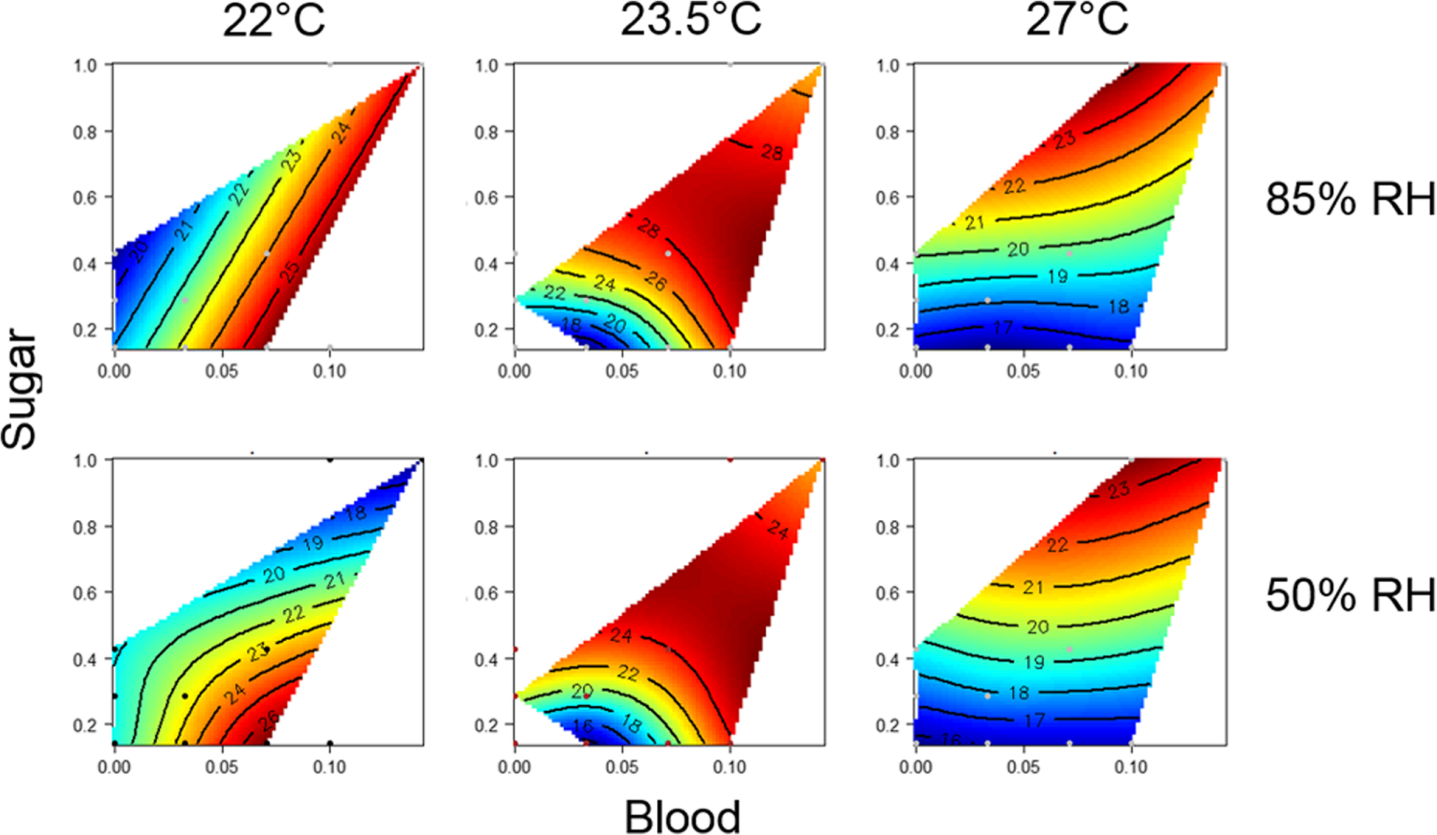
2D heat maps (thin-plate splines) of median lifespan in the six temperature/relative humidity (RH) combinations. *Red* regions in each response surface indicate greatest values for median lifespan. *Blue* regions indicate lowest values. Contour lines within the nutrient space display lifespan in days and reflect the specific combination of blood and sugar

To visualize these interactive effects, we used Nutritional Geometry to explore the effects of dietary blood and sugar on median lifespan (Fig. 5). Median lifespan varied significantly with both blood and sugar (Blood × Sugar interaction; *P* < 0.001), in accordance with the Cox regression analysis above, and the effect of these responses was dependent on RH and Temperature (*P* < 0.001, for both). At 23.5 °C and 27 °C, survival heat maps are similar between low and high RH. Median lifespan increased with temperature, and was greatest when both blood and sugar were highest. However, at the lower temperature of 22 °C, the surfaces show divergent landscapes. Here, the same nutrient combinations that extended lifespan of mosquitoes at 85% RH, reduced lifespan when subjected to lower RH. Under the cooler, dry conditions, maximal lifespans occurred when mosquitoes were fed blood once in 14 days and provided with 1 day of sugar per week (highly restricted diet). At 85% RH, sugar accessibility showed less influence on survival and maximal lifespans occurred when mosquitoes were fed blood either once every 7 or 14 days.

## Discussion

The dry season ecology presents a glaring gap in our understanding of malaria mosquitoes and disease transmission, including the seasonal changes in their physiology and behavior [9, 12, 15, 20–22]. Similar to previous studies on *An. arabiensis* [10, 11], recent studies have provided support for aestivation in *An. coluzzii* whereby the longevity of adult mosquitoes is extended for the duration of the dry season [6, 7, 20]. Yet, failure to find mosquitoes during the dry season in their shelters in the field, as well as to simulate aestivation in the laboratory, greatly hinder advances on this subject. Here we explored the effects of the nutritional regimes, combined with temperature and RH on longevity of adult *An. coluzzii*, focusing on the role of DR and P:C ratio. Longevity was treated as the ultimate measure of aestivation in this species. Our results revealed that changes in dietary regime, and specifically dietary restriction increased longevity substantially. This nutritional effect was considerably larger than the combined effect of temperature and RH. Moreover, we found evidence that DR increased longevity, whereas no support was detected for the effect of P:C ratio on mosquito longevity. Nonetheless, the increase in longevity measured in our experiments (overall mean = 20 d, maximum = 63 d) fell short from expected values based on aestivation (> 3 months). It is conceivable that different experimental conditions may have contributed, at least in part to our results; those possibly include mosquito crowding, oviposition deprivation, and ambient conditions. Although we have not successfully simulated aestivation under these laboratory conditions, we identified conditions that influence longevity, and thus are of importance for future studies on the induction and maintenance of aestivation under field and laboratory settings, as well as for understanding variation in vectorial capacity under wet season conditions.

### Longevity and climatic conditions

As expected, in both experiments lower temperature increased survival (Figs. 1a and 2, *P* < 0.001). Temperature has been shown to have an unambiguous effect on life span in various ectotherms, including *D. melanogaster* [77–80] and mosquitoes, including *Aedes albopictus* [81], *Culex quinquefasciatus* and *Cx. p. molestus* [82], *An. gambiae* (larvae) [83], and *An. stephensi* [84]. In our multivariate analysis, temperature was only significant in its interaction with RH, revealing a smaller effect of temperature difference at low RH possibly because of shorter longevity in low RH. Since RH during the DS varies between 15 and 25%, it suggests that mean longevity might be considerably shorter than 20 days regardless of temperature, unless mosquitoes remain in micro-climates of higher RH (refugia), and/or enter a different physiological state, as suspected.

### Longevity and diet

The effect of the diet regime was noticeably greater than that of temperature and RH, based on the estimates of the Chi-square and significance values, as well as the magnitude of the hazard odds ratios (Table 3). Variation within our experiments was probably elevated because our analysis is based on nutrients offered rather than nutrients consumed. This is probably a conservative effect, which was further reduced by observation that every blood meal resulted in > 90% feeding rate (although the quantity ingested could not be measured) and mosquitoes rapidly landed on sugar meals when offered. Nonetheless, the results revealed consistent and statistically significant effects of nutrients offered (Model 1 and 2, respectively), which are difficult to account for unless nutrients consumed were sufficiently correlated with nutrients offered. Based on the Nutritional Geometry Framework, which attributes lifespan increase to reduced protein over carbohydrate intake [64, 65], we subjected our mosquitoes to restrictive dietary sugar and blood while varying the P:C ratio between treatments (Table 3). The choice of a 7, 10 or 14-day interval between blood meals was based on the previously published blood-feeding protocol practiced at the LMVR insectary unit, where blood was offered once a week [73, 74]. A 14-day interval was considered by the authors to be a substantial dietary restrictive diet. It follows that a 10-day interval was the intermediate interval. Dietary sugar and blood prolonged longevity overall, but their effects were dependent on each other. Thus, addition of a blood meal to a sugar-deprived female reduced her hazard ratio (increased longevity) by 50%, whereas addition of a blood meal to a female having high dietary-sugar increased her hazard ratio by 950% (Table 3). Higher longevity was thus attained in groups with restricted access to blood and sugar, but not complete deprivation. Moreover, the effect of dietary blood was not linear throughout life and was found to be age-dependent, albeit age might have been confounded by increased dietary intake of sugar or blood over time. After accounting for the interaction with dietary sugar, dietary blood reduced the hazard ratio only in older mosquitoes, with the opposite result in younger ones (Table 3). The pronounced effect of DR on longevity was consistent with our prediction, but this was not the case for P:C ratio.

The generalized additive modelling (GAM) analysis showed that a one in 14-day blood meal resulted in greatest median lifespans despite changes to the physical environment, but that the effects of sugar on lifespan was dependent on both RH and temperature together (Fig. 5).

Accordingly, when sugar intake is higher than blood, as predicted for the dry season [15, 20] (and references therein), taking a blood meal could be detrimental for longevity (see in Fig. 5) [85], possibly explaining the low presence of mosquitoes indoors throughout most of the DS, interrupted only by 1–2 short periods of blood-feeding [7]. Together, the analyses above help visualize the complex interplay between physical and dietary variables, and their effects on lifespan of *An. coluzzii* (Fig. 5)*.*

During the Sahelian dry season *An. coluzzii* exhibited reduced flight activity [15], consistent with reduced foraging activity [20]. During the late dry season, as *An. coluzzii* are likely older, blood-feeding intensifies, fitting the finding that older (unlike young) mosquitoes benefit from ingesting blood. Taking blood meals during aestivation was reported previously. For example, in Sudan, the majority (> 77%) of the *An. arabiensis* during the DS were blood-fed, but refrained to oviposit (=gonotrophic dissociation) [10, 11], similarly to Sahelian *An. coluzzii* [9]. *Anopheles gambiae* (*s.l*.) from Kenya were observed to blood-feed although they were gravid during the dry season (José M. Ribeiro, pers. Comm.). In *Culiseta inornata* (Williston) (Diptera: Culicidae) in southern California, aestivating females took at least one blood meal and were parous [86]. The negligible effect of P:C ratio on longevity fits well with mosquito capacity to substitute sugar with blood. The observed reversal of dietary effects on longevity under low temperature and/or RH may suggest either a reduced capacity to digest blood (and/or sugar?) at lower temperatures, or provide an additional support for aestivation (and lifespan extension), occurring when temperature and RH are lowest. Whether availability of sources or other factors determined this choice remains unclear.

Previous studies on the effect of dietary blood and sugar on mosquito longevity provided conflicting results, with some claiming blood alone to be detrimental [87, 88] while others claiming the combination of the two is inferior to blood alone [89–93]. Much of the work involving the nutritional effects of blood and or sugar on mosquito survival were carried out on *Ae. aegypti* (see in references above), and may or may not bear relevance to *An. coluzzii*. These works include that of Harrington et al. [91], where females fed human blood and water had greater age-specific survival, reproductive output, and cumulative net replacement than cohorts fed blood supplemented with sugar or isoleucine-rich mouse blood with or without access to sugar. Others including Scott et al*.* found a fitness advantage to be gained by *Ae. aegypti* females frequently feeding on blood only, but also reported extended longevity in females fed on blood and sugar combined [90]. Straif & Beier [94] reported a 3-day difference in mean survival between blood and blood with sugar diets (16.2 and 19 days, respectively) in *An. gambiae*. They also found that older females (> 20 days) expressed both higher blood-feeding frequency and total blood feeds [94], supporting our results. In their study on mortality and reproduction of *Ae. aegypti*, Styer et al. [89] reported increased mortality in younger females which fed on blood. This phenomenon was reduced when blood was supplemented with sugar. The same study also showed increasing mean longevity in diets of blood only (~30 days), sugar only (~40 days) and blood with sugar (~53 days). A study on *An. gambiae* in Kenya also found an increasing rate of survival in females fed diets of blood, sugar or both, with mean survival in semi-field experiments of 5, 14 and 16 days, respectively [95]. Recently, Xue et al. [96] reported that *Ae. albopictus* females fed on a combination of blood and sugar survived longer (6–8-fold) than those fed on blood or blood and water. Considering the abundance of heme in the blood meal, its toxicity and potentially adverse effects on the mosquito, mechanisms of de-toxifying heme such as hemoglobin degradation within the peritrophic matrix may indeed be metabolically costly [85]. This suggests that blood might be differentially preferred over sugar when the mosquito can utilize the blood to maximize its fitness and benefit.

Some of the disparities aforementioned may be reconciled by our results showing the roles of DR and interaction between dietary sugar and blood in affecting longevity. This work, to the best of our knowledge, is the first considering DR and longevity in vectors of malaria in general, and the relationship of DR through Nutritional Geometry, and its relationship to aestivation specifically. Although our work is not definitive in its conclusions, it suggests a conceivable partial explanation to *An. coluzzii*’s ability to extend lifespan during the Sahelian dry season conditions. It additionally points to the dry season nutritional regimen of these vectors as a key to their extended survival, and thus as a potential bottle-neck for controlling vector populations using novel techniques such as toxic sugar baits [37].

The results presented here provide additional insights into the environmental factors shaping mosquito longevity and indirectly, vectorial capacity. Although these conditions failed to simulate aestivation in our laboratory strain (i.e. lifespan extension beyond 3 months), they suggest some role for the physical conditions (temperature, RH, and possibly photoperiod) as token stimuli to induce aestivation [20, 24, 97–99] and DR as a component for the maintenance of aestivation.

Presumably, additional factor(s) or a unique combination of values of the current parameters over one or more generations is required to simulate aestivating *An. coluzzii* under laboratory conditions. It is also possible, however, that our laboratory colony of *An. coluzzii*, which was established by offspring of six females in the end of the RS (2012), lack the complete genetic makeup required to express aestivation, even though it was established from a Sahelian population. If aestivation requires the combination of alleles from several polymorphic loci, it would be expressed only by a fraction of the population, which may have been missing in our six founder females. Alternatively, some of the required alleles have been lost during the colonization process. Indeed, if lifespan extension is linked with reduced reproduction ([9] and references above), these genotypes maybe selected against even after colonization. Reproduction, though a critical part of the diet-longevity equation, was beyond our experimental design as it would have required individual egg counts and opportunities to lay eggs in all treatments rather than only in wet season treatments since during the dry season there are no available larval sites (see Methods).

A follow-up study allowing the detection of the aestivator fraction of the population would therefore, require a higher diversity colony of recently obtained field material from the Sahel, with a larger mosquito sample per treatment, and would include scrutiny of reproduction to further explore the contribution of the P:C ratio.

## Conclusions

Dietary restriction promotes extended longevity in a wide variety of taxa. This study tested dietary restrictions’ ability to extend longevity in *Anopheles coluzzii* under different conditions simulating Sahelian seasonality. We showed extended longevity was a product of both lower temperature and higher RH, and a combination of dietary blood and sugar. Blood consumption increased longevity when sugar was restricted, but decreased it when sugar was available. This relationship was not linear throughout the life of the females, with blood meals reducing the hazard ratio in the older females. In dry-season conditions blood meals increased the hazard ratio when sugar was available. We found P:C ratio had a negligible effect on the risk of mortality. In this work, we have identified conditions that significantly extend longevity in malaria vectors in the laboratory setting, however, the extent of increase in longevity was insufficient to simulate aestivation.

## Additional file

Additional file 1: Figure S1. Low RH box showing: **a** Box open: (1) desiccant cup with (2) fan above it and (3) battery pack, (4) five half-gallon rearing cages and (5) a thermometer/hygrometer monitoring temperature and RH. **b** Sealed low RH box with top cover. (TIF 817 kb)

## Abbreviations

-2LL: -2 Log likelihood
AIC: Akaike information criterion
CI: Confidence interval
DR: Dietary restriction
DS: Dry season
GAM: Generalized additive modelling
HR: Hazard rate
IGF-I: Insulin-like growth factor 1
LMVR: Laboratory of malaria and vector research (NIH)
NGF: Nutritional geometry framework
P:C: Protein-to-carbohydrate ratio
RS: Rainy season
SIC: Schwarz information criterion
SOD: Superoxide dismutase
