# Supplementary figures and images for: The contribution of dietary restriction to extended longevity in the malaria vector Anopheles coluzzii
Source: Parasit Vectors. 2017 Mar 24;10:156. doi: 10.1186/s13071-017-2088-6 (PMC5366120; doi:10.1186/s13071-017-2088-6)

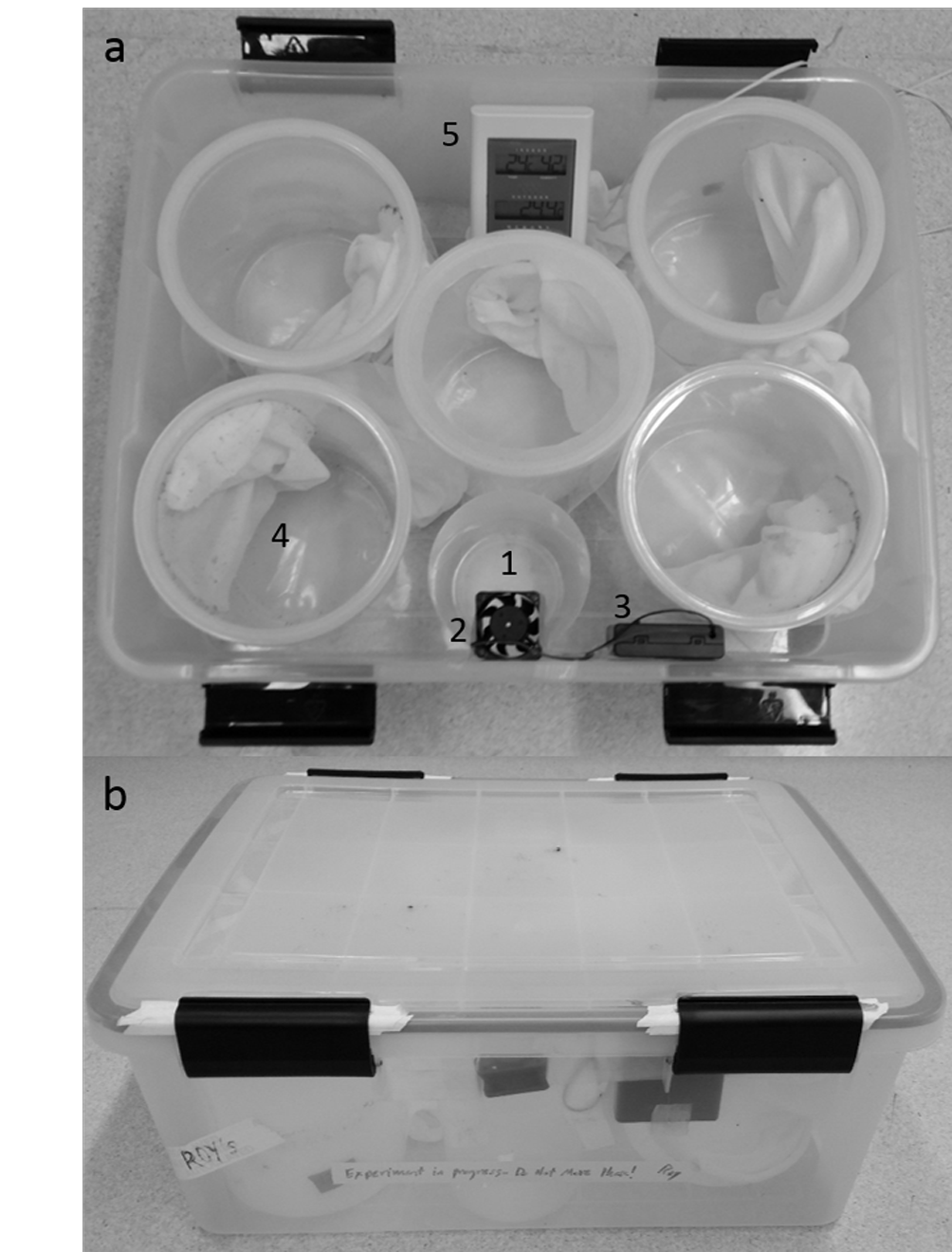

Supplement: Additional file 1: Figure S1. — Low RH box showing: a Box open: (1) desiccant cup with (2) fan above it and (3) battery pack, (4) five half-gallon rearing cages and (5) a thermometer/hygrometer monitoring temperature and RH. b Sealed low RH box with top cover. (TIF 817 kb) [file 13071_2017_2088_MOESM1_ESM.tif]
